# The role of the tryptophan metabolites in gut microbiota-brain axis and potential treatments: a focus on ischemic stroke

**DOI:** 10.3389/fphar.2025.1578018

**Published:** 2025-06-10

**Authors:** Na Qin, Xiaodi Xie, Rong Deng, Shiman Gao, Ting Zhu

**Affiliations:** ^1^ Institute of Neuroregeneration & Neurorehabilitation, Qingdao Medical College, Qingdao University, Qingdao, China; ^2^ Women and Children’s Hospital, Qingdao University, Qingdao, China

**Keywords:** tryptophan metabolites, gut microbiota, microbiota-gut-brain axis, ischemic stroke, kynurenine

## Abstract

Gut microbiota disturbances can elevate the risk of stroke by contributing to cerebrovascular events. Particularly, the gut tryptophan (TRP) metabolite is an essential mediator of the gut-brain axis. This review highlights the role of TRP metabolism in stroke, the influence of intestinal microbiomes on stroke pathology via TRP metabolism, and the gut-brain axis interactions. Recent studies indicate that various bioactive molecules produced via TRP metabolism can regulate various neurological functions and interrupt stroke pathophysiology. Moreover, the relationship between gut TRP metabolism and stroke development has been verified. TRP metabolism involves three pathways: kynurenine, 5-hydroxytryptamine, and indole, which potentially regulate post-stroke, may function as aryl hydrocarbon receptor agonists to modify neuronal excitotoxicity, and offer crucial targets for stroke treatment. This suggests that modulating TRP metabolite levels through various methods can enhance the prognosis of central nervous system diseases and restore microbiota-gut-brain axis functions.

## 1 Introduction

Stroke is an acute cerebrovascular disease and the second leading cause of death worldwide (Zhang et al., 2023; Hilkens et al., 2024; Xie et al., 2024b). Stroke is characterized by sudden interruption of cerebral blood flow resulting in neurological deficits (Xie et al., 2024a; Zhu et al., 2024). Despite numerous strategies and guidelines for stroke treatment, research on its precise etiology and pathology remains elusive.

Tryptophan (TRP) is an essential amino acid in humans that is crucial for the synthesis of various bioactive compounds and proteins and is obtained through diet. The TRP metabolism primarily involves three pathways: Kynurenine (KYN), 5-hydroxytryptamine (5-HT), and indoles (Cheong and Sun, 2018). KYN is the primary pathway for TRP metabolism, accounting for over 95% degradation of TRP bioactive compounds (Vécsei et al., 2013),which typically occurs in the liver, intestinal epithelial cells, and immune cells (Agus et al., 2018; Savitz, 2020). This pathway involves three key rate-limiting enzymes: indoleamine-2,3-dioxygenase 1 (IDO1), indoleamine-2,3-dioxygenase 2 (IDO2), and TRP-2,3-dioxygenase (TDO), and plays a role in neurotransmission, inflammation, and immune responses (Plitman et al., 2017). TRP in neurons of central nervous system (CNS) and enterochromaffin cells is converted to 5-hydroxytryptophan (5-HTP) and 5-HT to adapt to environmental changes, and plays a role in regulating intestinal motility, mood and cognitive function. (Turner et al., 2006). Unlike in the case of Try metabolism by the host enzyme, indoles and their derivatives are directly converted by gut microbes and modulate proinflammatory or anti-inflammatory cytokine expression to maintain intestinal homeostasis (Zhang J. et al., 2021). Accumulating evidence from multiple studies has demonstrated that TRP metabolism is closely associated with various neurological disorders, with its pathogenic role in stroke being particularly well-documented (Agus et al., 2018; Ma et al., 2020; Wu et al., 2024).

Preclinical and clinical studies have demonstrated the detailed mechanisms of the gut-brain axis from different perspectives, which indicate that the gut microbiota is essential for the regulation of human metabolic homeostasis and other physiological functions (Agirman et al., 2021; Mayer et al., 2022). Metabolites, such as short-chain fatty acids (SCFAs), TRP metabolites, and bile acids, are key mediators between intestinal microbiota and human health or disease (Xue et al., 2023). Studies have also demonstrated a strong link between gut microbiota and TRP metabolism in diseases through inflammatory or immune perspectives (Ding et al., 2021; Seo and Kwon, 2023). In addition to the indoles pathway, studies also addressed the importance of intestinal microbes in enhancing KYN pathway and affecting 5-HT pathway (Gao et al., 2020). All three pathways rely on intestinal microorganisms and are stimulated after supplementation with gut microbes (Clarke et al., 2013; Gao et al., 2020; Xue et al., 2023). Moreover, some homologous KYN pathway enzymes can be produced by specific gut bacteria and therefore widen the various KYN pathway metabolites (Vujkovic-Cvijin et al., 2013). Furthermore, previous studies have provided abundant evidence regarding the crucial roles of intestinal microbial flora in the pathological progression of nervous system diseases, which are partly realized through the regulation of TRP metabolism (Gheorghe et al., 2019; Chen et al., 2021; Liaqat et al., 2022).

Therefore, this review aims to provide a comprehensive summary of TRP metabolism and its significant impacts on stroke, with a particular emphasis on the roles of gut microbiota and the enhanced communication between the gut and brain. Specifically, we aimed to examine the impact of TRP metabolites and their derivatives on neurological diseases, particularly within the microbiota-gut-brain axis, and identify potential therapeutic targets. These discoveries will provide promising directions for future research on updating drugs for cerebral ischemia.

## 2 TRP metabolism and stroke

### 2.1 TRP metabolism pathways

A previous study observed improved TRP metabolism with reduced TRP levels in a post-stroke model, which evoked interest in the role of TRP in stroke pathology. After catalyzation by IDO or TDO to mediated compounds N-formylkynurenine (NFK), KYN is transformed and may undergo metabolism via one of the following directions: KYN converted to Kynurenic acid (KYNA) by KYN aminotransferases (KAT I–IV), anthranilic acid (AA) catalyzed by Kynureninase (KYNU) and 3-hydroxykynurenine (3-HK) by Kynurenine-3-monooxygenase (KMO). Additionally, 3-HK will be continually either catalyzed into 3-hydroxyanthranilic acid (3-HAA) and alanine by KYNU or xanthurenic acid (XA) by Kynurenine aminotransferase (KAT) (Lim et al., 2017). Furthermore, 3-HAA might convert to neurotoxic quinolinic acid (QA) catalyzed by 3-hydroxyanthranilate-3,4-dioxygenase (HAAO) (Xue et al., 2023). The KYNA pathway and other pathways have a protective compensatory mechanism; once KYNA levels increase, the 3-HAA/AA ratio decreases to fight secondary brain damage (Xue et al., 2023). In addition, 5-HTP is an intermediate compound in the 5-HT pathway, where it is catalyzed from TRP by Tryptophan hydroxylase (TPH) and converted to 5-HT by the interaction between aromatic L-amino acid decarboxylase and the cofactorpyridoxal-50-phosphate (Lim et al., 2017). Moreover, 5-HT can be synthesized in both the gut and brain, however, that synthesized in the gut cannot cross the blood-brain barrier (BBB) to enhance neurological function (Xue et al., 2023). The multiple biological functions of 5-HT in the brain are realized through combining different 5-HT receptors in relative cells and tissues (Xue et al., 2023). Furthermore, 5-HT can be converted to 5-hydroxyindole acetic acid (5-HIAA) and melatonin to perform other essential CNS functions, including mood regulation or the sleep-wake cycle (Xue et al., 2023). While indoles and its derivatives are mostly transformed by intestinal microorganisms in the gut (Agus et al., 2018). Tryptophan produces indole in the presence of tryptophanase (TnaA) from gut microbiota such as *E. coli* (Lee and Lee, 2010). Indole can be further metabolized to produce a variety of indole derivatives such as indole-3-acetic acid (IAA), indole-3-aldehyde (IAld), and indole-3-propionic acid (IPA) (Agus et al., 2018). Among them, tryptophan can be converted into indole-3-lactic acid (ILA) and IPA by *Clostridium sporogenes*, and IAld can also be converted from tryptophan by *Lactobacillus spp (* Roager and Licht, 2018; Gao et al., 2020).

### 2.2 Evidence of TRP metabolism in stroke

The pathogenesis of stroke has been studied in relation to primary and secondary periodic injuries. Following the primary insult of cerebral blood flow disruption or elevated intracranial pressure, secondary pathogenic mechanisms typically involve excitotoxicity, neuroinflammation, and oxidative stress (Ren et al., 2020; Zhao et al., 2020; Li et al., 2021). With the progression of cerebral ischemia, cell damage to the brain partly exacerbates the overactivation of N-methyl-D-aspartic acid (NMDA)-sensitive glutamate receptors, leading to an increased concentration of intracellular Ca^2+^ which in turn leads to the activation of destructive enzymes and production of reactive oxygen species (Vergun et al., 1999).

### 2.3 The role of TRP in post-stroke cognitive decline

With current standard of care involving timely treatments and proper management, stroke is becoming less severe and with patients demonstrating a longer survival. However, more than 30% of stroke survivors develop dementia within 5 years, leading to post-stroke cognitive impairment (PSCI) becoming an urgent public health issue (Kalaria et al., 2016). Cogo et al. (2021) explored the correlation between TRP metabolism and PSCI in diabetic ischemic mice, and reported that increased serum Quinidine (QUIN) levels and QUIN/KYNA ratios may result in degraded cognitive function (Cogo et al., 2021). Post-stroke inflammation results in an imbalance between cerebral pro- and anti-inflammatory cytokines and moving towards a severe inflammatory response, with the hypothesis that diabetes plays a role in increasing the inflammatory post-stroke status (Mangin et al., 2019). Such a status may increase the TRP conversion into KYN by IDO, although a previous study reported that serum IDO did not discriminate cognitive impairment in the post-stroke model compared with the control group. However, further studies verified the neurotoxic function of the increased cerebral and serum QUIN/KYNA ratio indicating increased activation of NMDA receptors, especially presenting degraded cognitive function on a spatial memory task (Cogo et al., 2021). Despite the need for future large prospective clinical studies to confirm the results in patients and to distinguish the effects of age and sex in mice, QUIN concentration and the QUIN/KYNA ratio provide reliable biomarkers for predicting PSCI.

## 3 TRP signaling pathway in stroke

### 3.1 TRP targeting the KYN pathway in stroke

L-TRP can be transferred from the peripheral intestinal tract across the BBB and be metabolized by IDO or TDO into L-KYN. In the KYN pathway, KYN, AA, 3-HK, and KYNA are converted in the brain and peripheral tissues, among which KYNA is mainly produced by astrocytes (Guillemin et al., 2001; Huang et al., 2023). Among them, only KYN, AA, 3-HK, and XA are able to cross the BBB and regulate neural functions; KYN can directly protect against damage induced by transient forebrain ischemia (Lee et al., 2015). An experiment revealed increased L-KYN levels and decreased L-TRP levels in the brain after MCAO (Fan et al., 2022). In the conversion processes, KYN positively affects the expression and activity of IDO. Thus, the KYN/TRP ratio is a valuable marker of IDO activity (Brochez et al., 2018). IDO-dependent TRP metabolism can modulate vessel atherosclerosis in cardiovascular diseases by increasing the levels of downstream metabolites, which can influence the apoptosis of innate and adaptive immune cells (Ketelhuth, 2019). One study reported that a higher KYN/TRP ratio is related to disease severity in cerebral ischemic injury, while another study suggested that reduced TRP indicates enhanced TRP metabolism, and increased IDO activity is correlated with stroke prognosis (Mo et al., 2014; Xue et al., 2023). As a vital noncompetitive NMDA glutamate receptor antagonist, KYNA plays a potential role in inhibiting excitotoxicity and neuroinflammation (Lim et al., 2017). Cozzi et al. found that KYN hydroxylase inhibitors upregulated KYNA and reduced infarct volume in a rat brain ischemia model (Cozzi et al., 1999).

Microglia expressing Kynurenine 3-Monooxygenase (KMO) promote the conversion of 3-HK to QA (Young et al., 2016). Moreover, 3-HK functions as a nerve agent which may result in neuronal degeneration and apoptosis by producing free radicals (Xue et al., 2023). QA is considered as a neurotoxic property for being an NMDAR agonist (Guillemin et al., 2003). QA converts to NAD^+^, participating energy metabolism (Xue et al., 2023). Increased brain QA was observed in transient ischemic attack (TIA) animal models via the activation of IDO, KYN and 3-HK, which probably contribute to stroke progression (Heyes and Nowak, 1990; Barattè et al., 1998). Moreover, QA/KYNA ratio has been investigated in animal experiments showing immune cell infiltration and increased severity of ischemic stroke as well as cognitive impairment (Cogo et al., 2021). However, María’ findings showed that KYN might not always exert neuroprotective effects, instead it may downregulate endogenous neuroprotective or anti-apoptotic pathways and participate in post-stroke brain damage (Cuartero et al., 2014b). It has been shown that the anti-inflammatory factor IL-4 downregulates the rate-limiting enzyme IDO, inhibiting KP activation and reducing Kyn production (Chen et al., 2024).

### 3.2 TRP targeting the 5-HT pathway in stroke

TRH1 transforms TRP into a precursor for 5-HT synthesis, which is upregulated in the brain tissue of MCAO animals 4 days after artery occlusion (Duan et al., 2018). Therefore, studies have suggested that TRH1 expression might be a risk factor for thrombosis (Boros et al., 2021). The role of 5-HT in innate and adaptive immunity has also been addressed. A recent study revealed that plasma 5-HT could stimulate monocytes and lymphocytes to regulate CNS function via cytokine secretion (McLean et al., 2007; Wu et al., 2019). Moreover, after stroke, the concentration of the 5-HT downstream molecule melatonin decreases. Thus, it may be a potential neuroprotective agent for improving prognosis (Huang et al., 2023). Multiple effects of melatonin have been observed, including antioxidation, anti-inflammation, anti-apoptosis, and the restoration of tissue function (Reiter et al., 2016). Melatonin can penetrate the BBB and reduce Glu toxicity (Alghamdi, 2018). Furthermore, melatonin can directly scavenge free radicals and indirectly inhibit oxidative enzymes, thereby facilitating antioxidant activity (Fox et al., 2007; Cavaleri, 2015). Additionally, melatonin displays anti-inflammatory effects by inhibiting NO, NF-κB signals and suggesting its role in the CNS (Simunkova et al., 2019). However, only few preclinical and clinical stroke studies have described the detailed effects of the 5-HT pathway, indicating a novel direction for research.

### 3.3 TRP targeting the indole pathway in stroke

Indole metabolism in the tryptophan metabolic pathway is strongly associated with stroke. Tryptophan metabolism produces indoles and their derivatives that are ligands for AhR. AhR is a highly conserved ligand-activated transcription factor that regulates immune differentiation and neuroinflammation (Agus et al., 2018). A study showed that AhR expression is upregulated after stroke, and that postoperative treatment of aged stroke mice by using AhR ligands such as IPA and IAld resulted in significant reductions in infarct volume and neurological defects, as well as amelioration of MG-mediated neuroinflammation (Peesh et al., 2025). In addition, It has been shown that IPA levels were reduced in middle cerebral artery occlusion mice, the structural richness of the gut flora as well as the area of cerebral infarction in mice was improved after IPA gavage and tube feeding (Xie et al., 2022). In addition, IPA may also exert antioxidant effects by mechanisms related to melatonin receptor binding in target cells and upregulation of peroxisome proliferator-activated receptor gamma-activated factor-1 α (PGC-1α) uncoupling protein 2 (UCP 2) expression (Li et al., 2022). IPA can also exert anti-inflammatory effects, and some experiments have shown that the level of pro-inflammatory factor IL-6 was decreased and the level of anti-inflammatory factor IL-10 was increased after IPA gavage (Xie et al., 2022). Overall, IPA may exert neuroprotective effects through antioxidant as well as modulating the levels of inflammatory factors. In addition, intestinal probiotics were positively correlated with stroke outcome, and IPA was also positively correlated with intestinal probiotics (Bhave et al., 2023). NLRP3 is an important sensor of innate immunity, and activation promotes IL-1β and IL-18 secretion to drive inflammation and cellular pyroptosis (Swanson et al., 2019). Microbial metabolites indole derivatives may inhibit NLRP3 inflammatory vesicles through activation of the AhR, reducing intestinal and systemic inflammation. It may also affect HPA axis function by modulating vagal or immune signaling, which can regulate the organism, stress immunity and metabolism (Rothhammer et al., 2018). A clinical trial showed that serum IPA levels were significantly lower in patients with acute cerebral infarction (ACI) than in healthy individuals, and thus IPA can be called an important indicator between ACI patients and healthy individuals (Li et al., 2024). Indole metabolites in the tryptophan metabolic pathway have important protective roles in stroke. These metabolites attenuate stroke injury through various mechanisms including modulation of intestinal flora and inhibition of inflammatory responses.

## 4 Correlation between gut microbiota and TRP metabolism in stroke

### 4.1 Gut microbiota and TRP metabolism

Although most TRP is ingested in the small intestine, a notable amount of TRP can be metabolized by the intestinal microbiota in the large intestine and participate in numerous physiological processes. TRP metabolism involves three direct and indirect pathways in the gastrointestinal tract. The gut microbiota can directly transform TRP into indoles and its derivates to act as ligands of the aryl hydrocarbon receptor (AhR) (Zelante et al., 2013). Moreover, approximately 90% of ingested TRP is degraded by immune and epithelial cells through the KYN pathway (Clarke et al., 2013; Vécsei et al., 2013). IDO1 is the only rate-limiting enzyme in the gut KP pathway and is immunoresponsive because of its similarity to innate immunity, in which microbial components such as LPS activate Toll-like receptors (TLRs) and initiate the KYN pathway (Kennedy et al., 2017). Additionally, TLRs, SCFAs, and specific molecules like H_2_O_2_ are mainly found to be involved in the modulation of central KYN pathway by gut microbes (Mu et al., 2016; Marin et al., 2017; Martin-Gallausiaux et al., 2018). Moreover, Gao et al. showed that KYNA could protect the mucosa and regulate immunity by binding to the G-protein-coupled receptor (GPR35) expressed in the gut epithelial and immune cells, indicating that metabolites of the KP pathway may exhibit specific effects in the gut (Gao et al., 2018). Notably, numerous studies have indicated that modulation of the KYN pathway, by altering the gut microbiota, might affect brain function, especially in terms of recognition and behavior (Gao et al., 2020). The gut is responsible for 90% of the 5-HT produced in the body, which is catalyzed by the TRP hydroxylase one enzyme (TPH1), and is mainly involved in the production of melatonin (Chen et al., 2011). Although 5-HT cannot cross the BBB under physiological conditions, the process of binding to various receptors can trigger 5-HT to function in the gastrointestinal tract affecting a wide range of human physiological processes (Mawe and Hoffman, 2013). On the contrary, gut microbiota also play a role in influencing CNS serotonergic neurotransmission by controlling peripheral TRP availability, whereas SCFA stimulation or inflammatory stimuli indirectly activate the KP pathway (Zhu et al., 2015; Brooks et al., 2016; Sun et al., 2016). Several reviews have indicated that several bacteria in the gut can directly produce indole and its derivatives, such as *Clostridium perfringens* converts tryptophan to tryptamine, ILA, and IPA; eptostreptococcus spp. converts tryptophan to IA and IPA; and *Lactobacillus* spp. converts tryptophan to IA via aromatic amino acid transaminase (ArAT) and indolylactic acid dehydrogenase (ILDH) (Agus et al., 2018). Indoles and their derivatives, such as IPA, IAA, IAld and indole-3-acetic acid (IAAld), in addition to acting on the gut microbiota, are absorbed into the blood circulation and affect brain function and behavior. In one study, acute mass production of indoles was mimicked by injecting indoles into the cecum of normal rats. This treatment resulted in a significant reduction in the locomotor behavior of the rats (Gao et al., 2020). Thus, gut microbiota-derived TRP metabolites exhibit multifaceted biological functions, including modulation of intestinal mucosal homeostasis, regulation of both innate and adaptive immune responses, and mediation of antioxidant and anti-inflammatory effects (Roager and Licht, 2018; Fan et al., 2022).

### 4.2 Gut microbiota and KYN pathway metabolism crosstalk in stroke

The gut-brain axis has been extensively studied for complicated signals and has revealed novel strategies targeting the gut microbiota, metabolites, and various ligands to ameliorate ischemic injury. Communication between the gut and brain occurs mainly via four mechanism: metabolism, immune signaling, endocrine signaling, and nerve conduction (Cox and Weiner, 2018). A previous review indicated dysbiosis of gut microbiota and interrupted TRP metabolism can be observed after cerebral ischemia through whole acute and chronic stages (Cuartero et al., 2014b; Cuartero et al., 2016). Thus, gut microbiota dysbiosis, such as the abnormal abundance of *Lactobacillus*, *Peptostreptococcus*, and *Akkermansia* in patients post-stroke, interferes with TRP metabolism (Pernomian et al., 2020; Yang et al., 2023).

### 4.3 Gut Microbiota-TRP metabolism-AhR-Th17/IL-17 signaling

**FIGURE 1:**
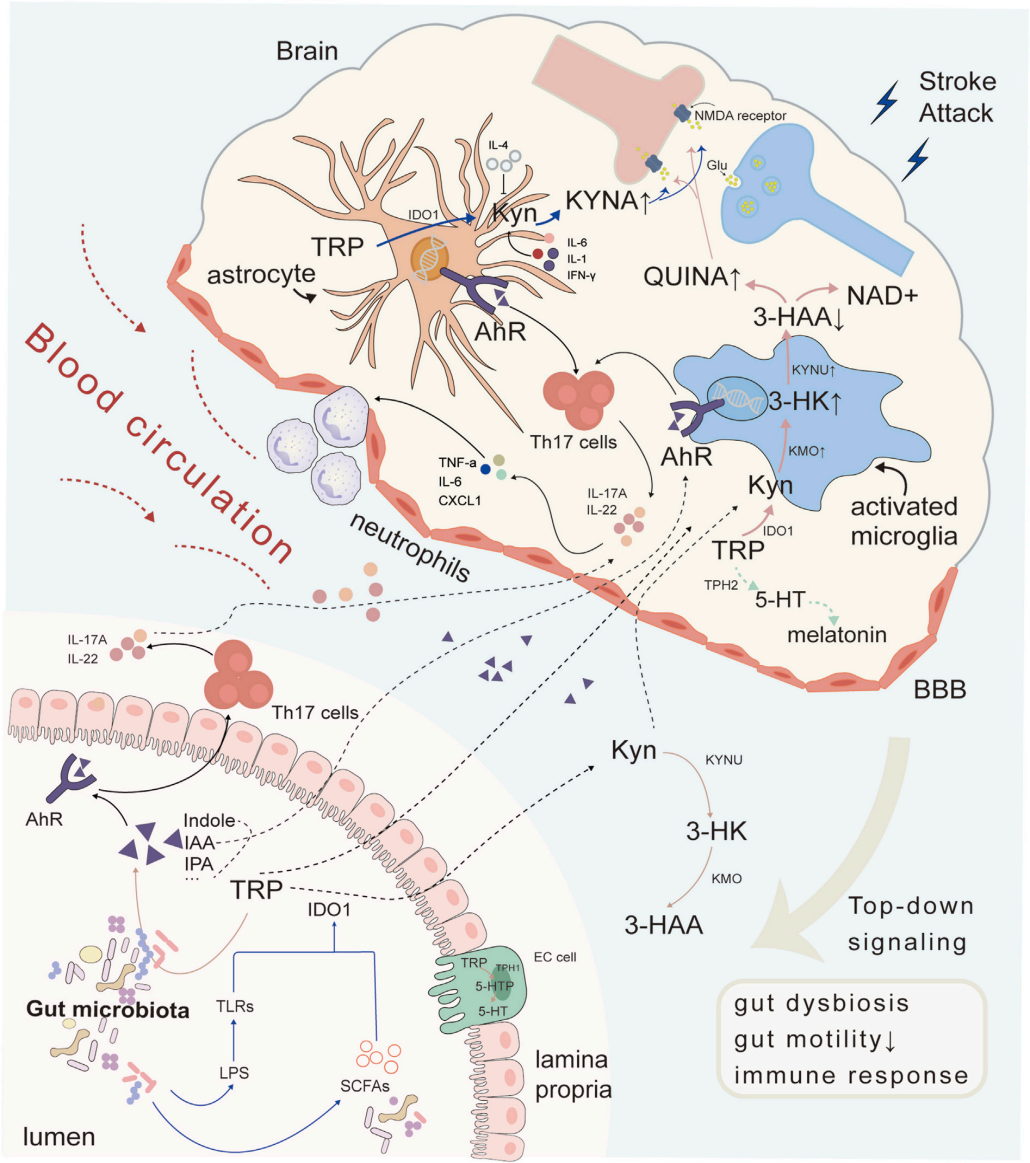
Crosstalk between gut microbiota and the brain through TRP metabolism after cerebral ischemia. Intestinal microbiota synthesized indole and its derivatives in TRP metabolism, which act as AhR receptor ligands to further induce Th17 cells to secrete cytokines IL-17A and IL-22 in intestinal cells. Metabolites and cytokines entered the blood circulation and entered the brain, participating in central immune inflammatory response together with glial AhR receptors, promoting peripheral neutrophils to pass through the BBB and exacerbating the inflammatory response. Other metabolites in TRP metabolism could also be influenced by gut microbiota. Both TRP and KYN could enter astrocytes and microglia through the blood to participate in brain TRP metabolism, producing KYNA and QUINA respectively, exerting neuroprotective or neurotoxic functions, and interfering with NMDARs. Abbreviations: TRP, tryptophan; 5-HT, 5-hydroxytryptamine; AhR, aryl hydrocarbon receptor; KYN, kynurenine; IDO1, indoleamine-2,3-dioxygenase 1; 5-HTP, 5-hydroxytryptophan; KYNA, kynurenic acid; KYNU, kynureninase; 3-HK, 3-hydroxykynurenine; KMO, kynurenine-3-monooxygenase; 3-HAA, 3-hydroxyanthranilic acid; NMDA, N-methyl-D-aspartic acid; TLRs, toll-like receptors; SCFAs, short-chain fatty acids; IPA, indole-3-propionic acid; IAA, indole-3-acid-acetic; IL-4, interleukin-4; QUINA, quinolinic acid; NAD+, nicotinamide adenine dinucleotide; TPH2, tryptophan hydroxylase 2; KMO, kynurenine 3-monooxygenase; IL-17A, interleukin-17A; IL-22, interleukin-22; IL-6, interleukin-6; Th17 cells, T helper 17 cell; TNF-α, tumor Necrosis Factor-alpha; CXCL1, Chemokine (C-X-C motif) ligand 1; EC cell, Embryonal carcinoma cell; LPS, Lipopolysaccharid.

The AhR has been suggested to be significantly activated during acute ischemic damage and subsequent neuroinflammation by TRP metabolites generated by gut microbes (Ma et al., 2020; Peesh et al., 2025). As a xenobiotic receptor (XR), AhR shuttles between the nucleus and cytoplasm to participate in the modulation of target genes expressed for cell proliferation, metabolism, and immune response (Mackowiak and Wang, 2016). In the KP pathway, KYN and KA were evaluated as AhR agonists in the pathological processes of cancer and the immune system (Liu et al., 2018). Additionally, 5-HT and its catabolites 5-HIAA are AhR ligands (Manzella et al., 2018). Moreover, most indole metabolites produced by gut microbes are recognized as AhR-selective agonists (Ma et al., 2020). Furthermore, AhR is widely expressed in the CNS neurons, endothelial cells and many glia including astrocytes and microglia (Rzemieniec et al., 2021). Gut TRP metabolites act as AhR agonist which facilitate its movement across BBB and the inhibition of nuclear factor-κB (NF-κB) via the activation of microglia and astrocytes. AhR not only interrupts chemokine production but also activates CNS resident myeloid cells and causes neurotoxicity to regulate CNS inflammation (Rothhammer et al., 2016). Thus, after stroke, crosstalk in the gut-brain axis may lead to gut microbiota dysbiosis and cause abnormal TRP metabolism, decreasing AhR agonists and inducing enhanced neuroinflammation through interactions with microglia and astrocytes (Fan et al., 2022). Alterations in the gut microbiota may also drive focal pro-inflammatory T helper cell differentiation and polarization, a process dependent on cytokines such as TGF-β, IL-6, and IL-23 (Ivanov et al., 2009). These differentiated cells subsequently migrate to the brain, where AhR activation induces the production of IL-17 and IL-22. Among them, Th17 cells differentiated from CD4 + Th cells can specifically produce IL-17, which plays a key role in exacerbating the post-stroke inflammatory response and mediating secondary neuronal injury (Veldhoen et al., 2008; Wang et al., 2024). Mechanistically, IL-17 binds to its receptor IL-17R, triggering activation of the adaptor protein Act1, which recruits TRAF6 to initiate downstream NF-κB and MAPK signaling pathways (Gaffen, 2009). This cascade promotes the secretion and expression of pro-inflammatory cytokines TNF-α, IL-6 and chemokines CXCL1/2 (Song et al., 2011), while concurrently enhancing neutrophil infiltration into the central nervous system, impairing the integrity of the BBB and stimulating the development of ischemic stroke (Benakis et al., 2016; Durgan et al., 2019; Sun et al., 2020; Zhang et al., 2021b). Therefore, gut microbiota-TRP metabolism-Th17/IL-17 signaling is a novel target for medical research, however, additional details regarding the multiple pathways are warranted (Figure 1).

## 5 TRP metabolism-related treatments

### 5.1 Indoles

Enteric microorganisms metabolize TRP into indoles and their derivatives. These metabolites can be absorbed into the circulatory system and contribute to immune function, metabolic processes, and neural communication within the “microbiota-gut-brain axis” through mechanisms dependent on the AhR as well as other pathways.

**FIGURE 2:**
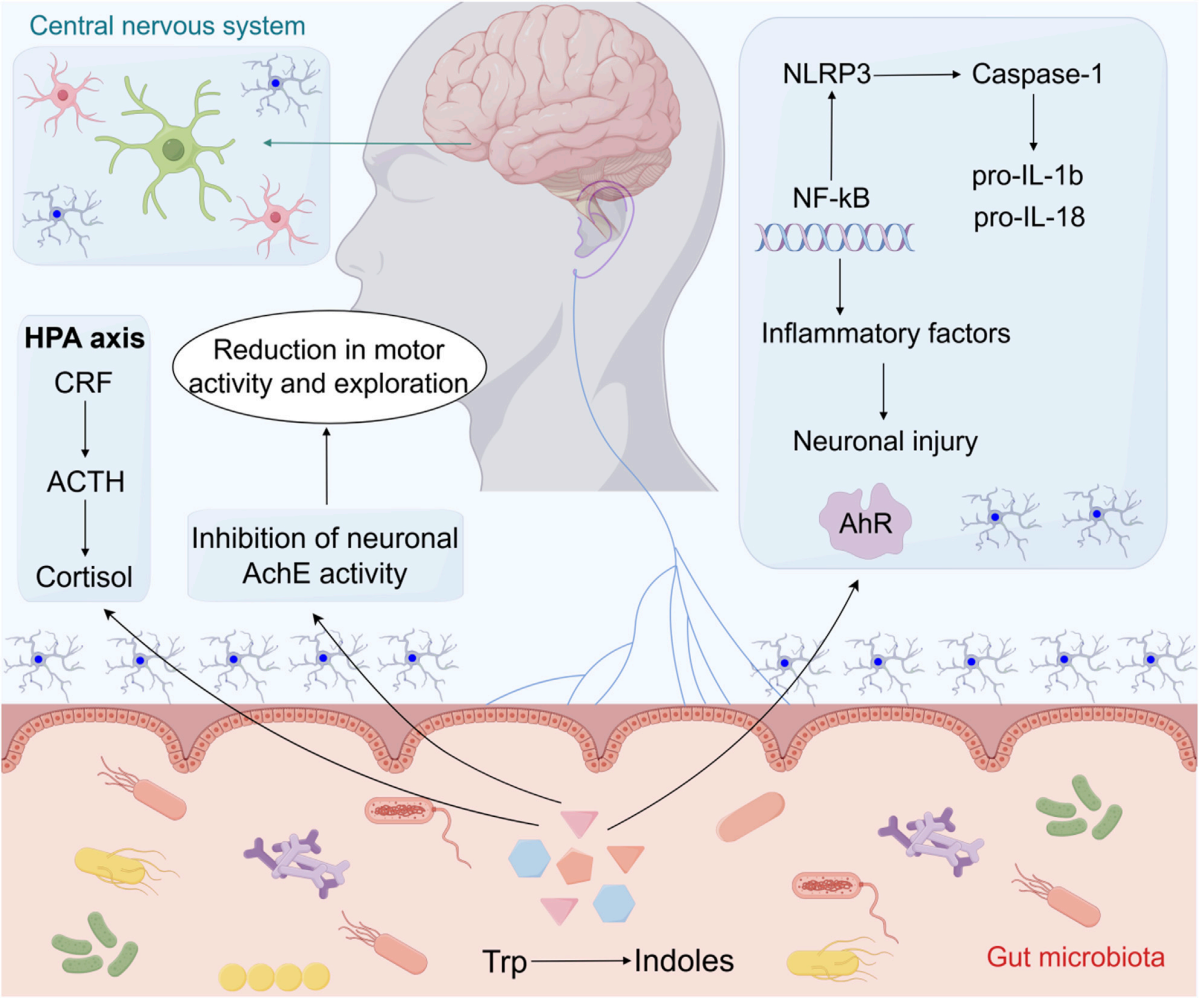
The effects of indoles on neurological disorders within the microbiota-gut-brain axis. By activating the AhR, indoles suppress neuroinflammation via inhibition of the NLRP3/NF-κB pathway and subsequent reduction of inflammatory cytokines (IL-1β, IL-18). Additionally, indoles inhibit AChE activity, affecting motor behavior, while modulation of the HPA axis (CRF-ACTH-cortisol) may contribute to stress-related behavioral changes. Abbreviations: HPA axis, the hypothalamic-pituitary-adrenal axis; CRF, Corticotropin releasing factor; ACTH, Adrenocorticotropic hormone; AchE, Acetylcholinesterase; NLRP3, NOD-like receptor thermal protein domin associated protein 3; NF-κB, Nuclear factor-κB; AhR, Aryl hydrocarbon receptor; TRP, Tryptophan; Pro-IL-1β, pro-interleukin-1β; Pro-IL-18, pro-interleukin-18.

Probiotic strains may enhance the diversity and prevalence of dominant gut microbial genera, thereby potentially serving as a therapeutic adjunct for neurological and neuropsychiatric disorders (Sanders et al., 2019). In a study focusing on patients with Parkinson’s disease (PD) with constipation, those receiving probiotics exhibited a significant reduction in gut transit time (GTT). Additionally, a notable increase in *g_Christensenella_*sp.*_Marseille-P2437* was observed in probiotic-treated mice, whereas *g_Eubacterium_oxidoreducens*_group, *g_Eubacterium_hallii*_group, and *s_Odoribacter_*sp.*_N54. MGS-14* were decreased (Du et al., 2022). Another study demonstrated that treatment with the probiotic *Bacteroides fragilis* corrected intestinal permeability in offspring with maternal immune activation and restored elevated levels of the pro-inflammatory cytokine IL-6 in the colon (Doenyas, 2018). Moreover, supplement probiotics showed reduced TNF-α and IFN-γ levels following chronic mild stress (Li et al., 2018). Probiotics have also been used in clinical trials to mitigate the adverse effects associated with gut microbial imbalance, potentially leading to the enrichment of indole-producing genera and their derivatives, such as *Lactobacillus* (Kelly et al., 2016). Additionally, research has indicated that *Lactobacillus* supplementation may enhance cognitive function and mood, and reduce aging-related inflammation in rodent models (Jeong et al., 2015; Chesnokova et al., 2016). It was found that Buqi-Huoxue-Tongnao (BHTD) reversed gut microbiota dysbiosis and upregulated tryptophan metabolism to enhance ILA synthesis to attenuate ischemic stroke (Liu et al., 2024). *Akkermansia muciniphila* (AKK) was found to be a promising probiotic that produces ILA with protective effects against ischemic stroke. ILA inhibited neuronal iron death by activating the aryl hydrocarbon receptor (AhR) and nuclear transcription factor Nrf2, upregulating SLC7A11 and GPX4 protein expression, and attenuating ischemic stroke-induced lipid peroxidation and intracellular iron accumulation (Wang et al., 2025) (Figure 2).

### 5.2 AhR

Indoles and their derivatives serve as ligands for AhR and are the principal targets of TRP metabolites in brain microvessels, demonstrating elevated expression of the AhR protein (Salminen, 2023). Consequently, the activation of AhR signaling disrupts vascular homeostasis in the brain, induces oxidative stress, stimulates inflammation, promotes cellular senescence, and enhances vascular wall calcification (Salminen, 2023). Moreover, the involvement of AhR ligands in the mitigation of learning and memory deficits has been corroborated in mouse models (Prinz et al., 2021). Activation of the AhR signaling pathway by endogenous ligands, including L-KYN and 6-Formylindolocarbazole (FICZ), as well as exogenous ligands, such as diosmin and indole-3-carbinol, enhances the expression and enzymatic activity of neprilysin in amyloid precursor protein/presenilin 1 (APP/PS1) transgenic mice. This activation effectively ameliorated cognitive impairment in these mice. Furthermore, TRP metabolites, including 5-hydroxyindoleacetic acid and Kynurenic acid, have shown promise in mitigating cognitive impairment and reducing amyloid-β (Aβ) burden in patients with mild cognitive impairment via activation of the AhR (Klein et al., 2018; Qian et al., 2021). In addition to targeting AhR, pharmacological agents that influence other components of the indole pathway may exhibit therapeutic potential. This hypothesis warrants further investigation through future research endeavors.

### 5.3 5-HT receptors

The gastrointestinal tract is colonized by trillions of bacteria that play a crucial role in regulating the host’s production of various signaling molecules, including 5-HT, hormones, and neurotransmitters. Approximately 90% of 5-HT is synthesized in the intestine (De Vadder et al., 2018). Activation of the 5-HT_4_ receptor (5-HT4R) within the enteric nervous system has been associated with adult neurogenesis and neuroprotection (De Vadder et al., 2018). Empirical evidence has confirmed the expression of 5-HT4R in the enteric nervous system, particularly within myenteric neurons, and has demonstrated that this expression is contingent upon the presence of a gut microbiota (De Vadder et al., 2018). Moreover, Prucalopride, AT-7505, and Velusetrag are three novel 5-HT_4_ receptor agonists that have been evaluated for the treatment of chronic constipation, with prucalopride demonstrating efficacy in three multicenter studies (Quigley and Craig, 2012). The 5-HT_3_ receptor is not only present in the gastrointestinal epithelium (Glatzle et al., 2002; Walstab et al., 2014), but also plays a role in mediating intestinal secretion via a non-neuronal pathway (Budhoo et al., 1996). Additionally, the gut microbiota has been shown to enhance 5-HT biosynthesis through the action of SCFAs, which may subsequently influence 5-HT_3_ receptor expression. Furthermore, 5-HT_3_ receptor antagonists influence the transit time and inhibit visceral pain, as demonstrated in animal and human studies (McLean et al., 2007). Consequently, these compounds have been used to treat diarrhea-predominant irritable bowel syndrome (IBS). Particularly, romosetron, a novel 5-HT_3_ receptor antagonist, has been shown to ameliorate global symptoms in both female and male patients with IBS without any significant adverse effects. This compound has now received approval for use in Japan (Chey et al., 2011). In addition, it has been found that melatonin, as a product of the 5-HT pathway of tryptophan metabolism, stimulates the proliferation of specific beneficial bacteria, including *Enterobacter*, the *Bacteroidales S24-7 group*, *Prevotella 9*, Ruminococcaceae, and Lachnospiraceae. These bacteria collectively established a favorable co-occurrence pattern within the intestinal microecology. This shift enhanced gut homeostasis and strengthened the intestinal barrier function, which in turn mitigated both brain and gut injury (Lian et al., 2023).

### 5.4 Diet

Gut microbiota has been identified as a critical factor in the regulation of brain processes and behavior. Diet plays a significant role in shaping the composition of the gut microbiota throughout the lifespan (Berding et al., 2021). Research has indicated that a high-fat diet may impair the microbial TRP degradation pathway (Krishnan et al., 2018). Furthermore, increased carbohydrate availability has been shown to enhance intestinal serotonin synthesis (Kashyap et al., 2013). TRP, an essential amino acid in the human body, is obtained via dietary intake. Foods rich in TRP include fish, poultry, cereals, and dairy products (Fang et al., 2022). The TRP-enriched diet demonstrated neurotherapeutic potential by augmenting the dietary TRP intake and leveraging the conversion of TRP by the gut microbiota to regulate indole levels. Researchers observed that a diet rich in TRP (0.6% tryptophan (TRP, w/w) significantly ameliorated neuroinflammation, enhanced BDNF expression, and improved mitochondrial energy metabolism in the brains of mice subjected to chronic unpredictable mild stress (CUMS). Additionally, TRP supplementation may preferentially shift TRP metabolism towards the serotonin pathway in CUMS-treated mice (Wang et al., 2022). Serum-targeted metabolomics and 16S rRNA sequencing further corroborated the potential role of the microbiota-gut-brain axis in modulating depressive-like behavioral dysfunction in the context of a TRP-rich diet. However, excessive TRP intake can result in the accumulation of KYN and indole metabolites, activation of the AhR pathway, and subsequent kidney injury (Hu et al., 2023). Given the alterations in the human gut microbiome following nervous system injury (Kigerl et al., 2016), dietary regulation may represent a straight forward yet efficacious approach for the treatment of neurological and neuropsychiatric disorders (Table 1).

**TABLE 1:** Tryptophan metabolism-related treatments and different neurological diseases: Preclinical studies.

Treatments	Disease	Subjects	Samples	Results	References
Probiotics	Parkinson’s disease	Parkinson’s disease patients with constipation Probiotic-treated mice	Fresh stool samples	↓BSS score (0.65 ± 0.93 vs. − 0.17 ± 0.94, P = 0.004), PAC-SYM score (4.09 ± 6.31 vs. − 1.83 ± 4.14, P < 0.001), PAC-QOL score (10.65 ± 16.53 vs. 0.57 ± 12.82, P = 0.042), and degree of defecation effort score (1.00 ± 0.80 vs. 0.00 ± 0.30, P < 0.001) ↑g_Christensenella_sp._Marseille-P2437 ↓g_Eubacterium_oxidoreducens_group, g_Eubacterium_hallii_group and s_Odoribacter_sp. _N54.MGS-14	Du et al. (2022)
Probiotics	Chronic mild stress	Chronic mild stress mouse model Old male C57BL/6 mice	Hippocampus sample Cecal contents	↓Anxiety- and depressive-like behaviors ↑*Lactobacillus* abundance Reverse the immune changes in the hippocampus	Li et al. (2018)
Probiotics	Depression	Patients with major depression Microbiota-depleted rats	Fecal samples	↓Adverse effects associated with gut microbial imbalance ↑Indole-producing genera and their derivatives, such as *Lactobacillus*	Kelly et al. (2016)
*Lactobacillus* pentosus var. plantarum C29	Memory impairment	Aged Fischer 344 rats Male C57BL/6 mice	Hippocampus samples Peritoneal macrophages	↓The expression of p16, cyclooxygenase-2, and inducible nitric oxide synthase Activate Akt, mTOR, and NF-κB in the hippocampus	Jeong et al. (2015)
AhR agonists	Alzheimer’s disease	APP/PS1 mice N2a cells	Brain tissues Cells sample	↑NEP expression and enzyme activity	Qian et al. (2021)
5-hydroxyindolacetic acid (5-HIAA)	Alzheimer’s disease	Transgenic APPSWE mouse model Human neuroblastoma SH-SY5Y cells and human neuroblastoma SH-SY5Y-APPwt cells	Brain tissue Cells sample	↑Memory performance ↑NEP level *in vivo* and in neuroblastoma cells	Klein et al. (2018)
Indole-3-propionic acid (IPA)	Sepsis-associated encephalopathy (SAE)	Specific pathogen-free male C57BL/6 mice Pure primary microglia	Mouse feces Cortex and cells sample	↓CLP-induced anxiety and spatial memory impairment in septic mice ↓NLRP3 inflammasome activation and IL-1β secretion in lipopolysaccharide-stimulated microglia	Fang et al. (2022)
Tryptophan-rich diet (0.6% tryptophan w/w)	Chronic unpredictable mild stress (CUMS)	Male C57BL/6 mice	Brain and gut tissue Serum samples	↓Depression- and anxiety-like behaviors ↑ Neuroinflammation and mitochondrial energy metabolism ↑The expression of BDNF ↓Stress-induced gut barrier damage ↓Inflammatory responses in the colon	Wang et al. (2022)

## 6 Discussion

TRP metabolites are essential for protein biosynthesis and mainly participate in neuronal construction and maintenance (Huang et al., 2023). These metabolites participate in the entire process of neurological diseases such as depression, schizophrenia, AD, and cerebral ischemic injury. Among them, it has been noted that the inflammatory response due to stroke-induced brain injury is associated with activation of kynurenine metabolism of tryptophan metabolism (Brouns et al., 2010). In addition, a significant correlation was found between the decrease in the 3HAA:AA ratio and infarct volume (Darlington et al., 2007). IDO1, one of the key enzymes of the kynurenine metabolic pathway, has increased activity after stroke onset and is associated with an increased risk of death. In this study, the results showed an approximately six-fold increase in IDO activity after stroke and a more than two-fold increase in both plasma kynurenine accumulation and tryptophan consumption. However, treatment with the IDO inhibitor 1-MT reduced mean IDO activity by >50% (Jackman et al., 2011). AhR has an important role in stroke, and its activation can aggravate ischemic injury and promote inflammatory response. In mice, AhR is overexpressed in the ischemic core infarct, peri-infarct, and cortical regions, and its activation inhibits the cAMP response element binding protein (CREB) signaling pathway, which in turn inhibits the expression of neurotrophic factors (e.g., brain-derived neurotrophic factor (BDNF)) and exacerbates neuronal injury. (Cuartero et al., 2014a). Therefore, the regulation of the tryptophan metabolic pathway is of great significance in relation to the onset and progression of ischemic stroke.

Cross talk in the gut-brain axis has verified that stroke may cause intestinal microbial dysbiosis, abnormal intestinal motility, destroyed gut barrier, failed response to stress, and systemic infection (Xie et al., 2024a). In contrast, these processes can change the abundance of gut microbiota, abnormal immune cell migration, and result in an imbalance in inflammatory status, which induces severe ischemic stroke and exacerbates prognosis (Martin et al., 2018). According to related studies, ischemic stroke causes gut microbiota disruption, and the phyla Bacteroidetes, the family Prevotellaceae, and the genera Alloprevotella show a significant increase in abundance after stroke onset, while the phyla Firmicutes, the family Lachnospiraceae, and the genera Roseburia significantly decreased in abundance (Zhang et al., 2022). Furthermore, studies focusing on adult hippocampus neurogenesis, which is also regarded as potential therapeutic strategy for neural regeneration, have verified that indole-AhR signaling represents one mechanism of gut microbes (Wei et al., 2021).

Thus, this review summarizes novel insights into the relationship between gut TRP metabolism in microbes and its influence on stroke by targeting the gut-brain axis and partially ignores TRP metabolism in the pathophysiology of intracerebral hemorrhage (ICH), which warrants further studies to explore the role of TRP in the preclinical and clinical status of ICH. Since previous studies have suggested several targets to improve prognosis of stroke including inhibitors of TDO, KMO, NMDAR, oral melatonin which shows efficacy of reducing the severity of stroke and improve cognitive symptoms and AhR is thought to be a potential therapeutic target for tightly participating in “gut microbiota-TRP-brain” axis in ischemia stroke treatment. Nevertheless, further research is required to identify molecules involved in the multiple directions of stroke treatment focusing on TRP metabolism (Sadanandan et al., 2020; MartInez-Coria et al., 2021).

## Abbreviations

TRP, Tryptophan; 5-HT, 5-hydroxytryptamine; AhR, Aryl hydrocarbon receptor; KYN, Kynurenine; IDO1, Indoleamine-2,3-dioxygenase 1; TDO, Trp-2,3-dioxygenase; 5-HTP, 5-hydroxytryptophan; KYNA, Kynurenic acid; AA, Anthranilic acid; KYNU, Kynureninase; 3-HK, 3-hydroxykynurenine; KMO, Kynurenine-3-monooxygenase; 3-HAA, 3-hydroxyanthranilic acid; XA, Xanthurenic acid; QA, Quinolinic acid; BBB, Blood-brain barrier; NMDA, N-methyl-D-aspartic acid; TIA, Transient ischemic attack; TLRs, Toll-like receptors; SCFAs, Short-chain fatty acids; IPA, Indole-3-propionic acid; IAA, Indole-3-acid-acetic; IAld, Indole-3-aldehyde; IAAld, Indole-3-acetaldehyde; NF-κB, Nuclear factor-κB; PD, Parkinson’s disease.

## References

[B1] Agirman G. Yu K. B. Hsiao E. Y. (2021). Signaling inflammation across the gut-brain axis. Science 374 (6571), 1087–1092. 10.1126/science.abi6087 34822299

[B2] Agus A. Planchais J. Sokol H. (2018). Gut microbiota regulation of tryptophan metabolism in health and disease. Cell Host Microbe 23 (6), 716–724. 10.1016/j.chom.2018.05.003 29902437

[B4] Alghamdi B. S. (2018). The neuroprotective role of melatonin in neurological disorders. J. Neurosci. Res. 96 (7), 1136–1149. 10.1002/jnr.24220 29498103 PMC6001545

[B5] Barattè S. Molinari A. Veneroni O. Speciale C. Benatti L. Salvati P. (1998). Temporal and spatial changes of quinolinic acid immunoreactivity in the gerbil hippocampus following transient cerebral ischemia. Brain Res. Mol. Brain Res. 59 (1), 50–57. 10.1016/s0169-328x(98)00136-3 9729272

[B6] Benakis C. Brea D. Caballero S. Faraco G. Moore J. Murphy M. (2016). Commensal microbiota affects ischemic stroke outcome by regulating intestinal γδ T cells. Nat. Med. 22 (5), 516–523. 10.1038/nm.4068 27019327 PMC4860105

[B7] Berding K. Vlckova K. Marx W. Schellekens H. Stanton C. Clarke G. (2021). Diet and the microbiota-gut-brain Axis: sowing the seeds of good mental health. Adv. Nutr. 12 (4), 1239–1285. 10.1093/advances/nmaa181 33693453 PMC8321864

[B8] Bhave V. M. Ament Z. Patki A. Gao Y. Kijpaisalratana N. Guo B. (2023). Plasma metabolites link dietary patterns to stroke risk. Ann. Neurol. 93 (3), 500–510. 10.1002/ana.26552 36373825 PMC9974740

[B9] Boros F. A. Maszlag-Török R. Szűcs M. Annus Á. Klivényi P. Vécsei L. (2021). Relationships of ischemic stroke occurrence and outcome with gene variants encoding enzymes of tryptophan metabolism. Biomedicines 9 (10), 1441. 10.3390/biomedicines9101441 34680558 PMC8533114

[B10] Brochez L. Meireson A. Chevolet I. Sundahl N. Ost P. Kruse V. (2018). Challenging PD-L1 expressing cytotoxic T cells as a predictor for response to immunotherapy in melanoma. Nat. Commun. 9 (1), 2921. 10.1038/s41467-018-05047-1 30050132 PMC6062523

[B11] Brooks A. K. Lawson M. A. Rytych J. L. Yu K. C. Janda T. M. Steelman A. J. (2016). Immunomodulatory factors galectin-9 and interferon-gamma synergize to induce expression of rate-limiting enzymes of the kynurenine pathway in the mouse Hippocampus. Front. Immunol. 7, 422. 10.3389/fimmu.2016.00422 27799931 PMC5065983

[B12] Brouns R. Verkerk R. Aerts T. De Surgeloose D. Wauters A. Scharpé S. (2010). The role of tryptophan catabolism along the kynurenine pathway in acute ischemic stroke. Neurochem. Res. 35 (9), 1315–1322. 10.1007/s11064-010-0187-2 20490917

[B13] Budhoo M. R. Harris R. P. Kellum J. M. (1996). The role of the 5-HT4 receptor in Cl-secretion in human jejunal mucosa. Eur. J. Pharmacol. 314 (1-2), 109–114. 10.1016/s0014-2999(96)00474-8 8957225

[B14] Cavaleri F. (2015). Review of Amyotrophic Lateral Sclerosis, Parkinson's and Alzheimer's diseases helps further define pathology of the novel paradigm for Alzheimer's with heavy metals as primary disease cause. Med. Hypotheses 85 (6), 779–790. 10.1016/j.mehy.2015.10.009 26604027

[B15] Chen C. Q. Fichna J. Bashashati M. Li Y. Y. Storr M. (2011). Distribution, function and physiological role of melatonin in the lower gut. World J. Gastroenterol. 17 (34), 3888–3898. 10.3748/wjg.v17.i34.3888 22025877 PMC3198018

[B16] Chen L. M. Bao C. H. Wu Y. Liang S. H. Wang D. Wu L. Y. (2021). Tryptophan-kynurenine metabolism: a link between the gut and brain for depression in inflammatory bowel disease. J. Neuroinflammation 18 (1), 135. 10.1186/s12974-021-02175-2 34127024 PMC8204445

[B17] Chen W. Tian Y. Gou M. Wang L. Tong J. Zhou Y. (2024). Role of the immune-kynurenine pathway in treatment-resistant schizophrenia. Prog. Neuropsychopharmacol. Biol. Psychiatry 130, 110926. 10.1016/j.pnpbp.2023.110926 38147973

[B18] Cheong J. E. Sun L. (2018). Targeting the Ido1/TDO2-KYN-AhR pathway for cancer immunotherapy - challenges and opportunities. Trends Pharmacol. Sci. 39 (3), 307–325. 10.1016/j.tips.2017.11.007 29254698

[B19] Chesnokova V. Pechnick R. N. Wawrowsky K. (2016). Chronic peripheral inflammation, hippocampal neurogenesis, and behavior. Brain Behav. Immun. 58, 1–8. 10.1016/j.bbi.2016.01.017 26802985 PMC4956598

[B20] Chey W. D. Maneerattaporn M. Saad R. (2011). Pharmacologic and complementary and alternative medicine therapies for irritable bowel syndrome. Gut Liver 5 (3), 253–266. 10.5009/gnl.2011.5.3.253 21927652 PMC3166664

[B21] Clarke G. Grenham S. Scully P. Fitzgerald P. Moloney R. D. Shanahan F. (2013). The microbiome-gut-brain axis during early life regulates the hippocampal serotonergic system in a sex-dependent manner. Mol. Psychiatry 18 (6), 666–673. 10.1038/mp.2012.77 22688187

[B22] Cogo A. Mangin G. Maïer B. Callebert J. Mazighi M. Chabriat H. (2021). Increased serum QUIN/KYNA is a reliable biomarker of post-stroke cognitive decline. Mol. Neurodegener. 16 (1), 7. 10.1186/s13024-020-00421-4 33588894 PMC7885563

[B23] Cox L. M. Weiner H. L. (2018). Microbiota signaling pathways that influence neurologic disease. Neurotherapeutics 15 (1), 135–145. 10.1007/s13311-017-0598-8 29340928 PMC5794708

[B24] Cozzi A. Carpenedo R. Moroni F. (1999). Kynurenine hydroxylase inhibitors reduce ischemic brain damage: studies with (m-nitrobenzoyl)-alanine (mNBA) and 3,4-dimethoxy-[-N-4-(nitrophenyl)thiazol-2yl]-benzenesulfonamide (Ro 61-8048) in models of focal or global brain ischemia. J. Cereb. Blood Flow. Metab. 19 (7), 771–777. 10.1097/00004647-199907000-00007 10413032

[B25] Cuartero M. I. Ballesteros I. de la Parra J. Harkin A. L. Abautret-Daly A. Sherwin E. (2014a). L-Kynurenine/Aryl hydrocarbon receptor pathway mediates brain damage after experimental stroke. Circulation 130 (23), 2040–2051. 10.1161/CIRCULATIONAHA.114.011394 25359166

[B26] Cuartero M. I. Ballesteros I. de la Parra J. Harkin A. L. Abautret-Daly A. Sherwin E. (2014b). L-kynurenine/aryl hydrocarbon receptor pathway mediates brain damage after experimental stroke. Circulation 130 (23), 2040–2051. 10.1161/circulationaha.114.011394 25359166

[B27] Cuartero M. I. de la Parra J. García-Culebras A. Ballesteros I. Lizasoain I. Moro M. (2016). The kynurenine pathway in the acute and chronic phases of cerebral ischemia. Curr. Pharm. Des. 22 (8), 1060–1073. 10.2174/1381612822666151214125950 25248805 PMC4972938

[B28] Darlington L. G. Mackay G. M. Forrest C. M. Stoy N. George C. Stone T. W. (2007). Altered kynurenine metabolism correlates with infarct volume in stroke. Eur. J. Neurosci. 26 (8), 2211–2221. 10.1111/j.1460-9568.2007.05838.x 17892481

[B29] De Vadder F. Grasset E. Mannerås Holm L. Karsenty G. Macpherson A. J. Olofsson L. E. (2018). Gut microbiota regulates maturation of the adult enteric nervous system via enteric serotonin networks. Proc. Natl. Acad. Sci. U. S. A. 115 (25), 6458–6463. 10.1073/pnas.1720017115 29866843 PMC6016808

[B30] Ding M. Lang Y. Shu H. Shao J. Cui L. (2021). Microbiota-gut-brain Axis and epilepsy: a review on mechanisms and potential therapeutics. Front. Immunol. 12, 742449. 10.3389/fimmu.2021.742449 34707612 PMC8542678

[B31] Doenyas C. (2018). Gut microbiota, inflammation, and probiotics on neural development in autism spectrum disorder. Neuroscience 374, 271–286. 10.1016/j.neuroscience.2018.01.060 29427656

[B32] Du Y. Li Y. Xu X. Li R. Zhang M. Cui Y. (2022). Probiotics for constipation and gut microbiota in Parkinson's disease. Park. Relat. Disord. 103, 92–97. 10.1016/j.parkreldis.2022.08.022 36087572

[B33] Duan X. Gan J. Xu F. Li L. Han L. Peng C. (2018). RNA sequencing for gene expression profiles in a rat model of middle cerebral artery occlusion. Biomed. Res. Int. 2018, 2465481. 10.1155/2018/2465481 30533429 PMC6247679

[B34] Durgan D. J. Lee J. McCullough L. D. Bryan R. M. Jr. (2019). Examining the role of the microbiota-gut-brain Axis in stroke. Stroke 50 (8), 2270–2277. 10.1161/strokeaha.119.025140 31272315 PMC6646086

[B35] Fan X. Wang S. Hu S. Yang B. Zhang H. (2022). Host-microbiota interactions: the aryl hydrocarbon receptor in the acute and chronic phases of cerebral ischemia. Front. Immunol. 13, 967300. 10.3389/fimmu.2022.967300 36032153 PMC9411800

[B36] Fang H. Wang Y. Deng J. Zhang H. Wu Q. He L. (2022). Sepsis-induced gut dysbiosis mediates the susceptibility to sepsis-associated encephalopathy in mice. mSystems 7 (3), e0139921. 10.1128/msystems.01399-21 35642838 PMC9239149

[B37] Fox J. H. Kama J. A. Lieberman G. Chopra R. Dorsey K. Chopra V. (2007). Mechanisms of copper ion mediated Huntington's disease progression. PLoS One 2 (3), e334. 10.1371/journal.pone.0000334 17396163 PMC1828629

[B38] Gaffen S. L. (2009). Structure and signalling in the IL-17 receptor family. Nat. Rev. Immunol. 9 (8), 556–567. 10.1038/nri2586 19575028 PMC2821718

[B39] Gao J. Xu K. Liu H. Liu G. Bai M. Peng C. (2018). Impact of the gut microbiota on intestinal immunity mediated by tryptophan metabolism. Front. Cell Infect. Microbiol. 8, 13. 10.3389/fcimb.2018.00013 29468141 PMC5808205

[B40] Gao K. Mu C. L. Farzi A. Zhu W. Y. (2020). Tryptophan metabolism: a link between the gut microbiota and brain. Adv. Nutr. 11 (3), 709–723. 10.1093/advances/nmz127 31825083 PMC7231603

[B41] Gheorghe C. E. Martin J. A. Manriquez F. V. Dinan T. G. Cryan J. F. Clarke G. (2019). Focus on the essentials: tryptophan metabolism and the microbiome-gut-brain axis. Curr. Opin. Pharmacol. 48, 137–145. 10.1016/j.coph.2019.08.004 31610413

[B42] Glatzle J. Sternini C. Robin C. Zittel T. T. Wong H. Reeve J. R. Jr. (2002). Expression of 5-HT3 receptors in the rat gastrointestinal tract. Gastroenterology 123 (1), 217–226. 10.1053/gast.2002.34245 12105850

[B43] Guillemin G. J. Kerr S. J. Smythe G. A. Smith D. G. Kapoor V. Armati P. J. (2001). Kynurenine pathway metabolism in human astrocytes: a paradox for neuronal protection. J. Neurochem. 78 (4), 842–853. 10.1046/j.1471-4159.2001.00498.x 11520905

[B44] Guillemin G. J. Smith D. G. Smythe G. A. Armati P. J. Brew B. J. (2003). Expression of the kynurenine pathway enzymes in human microglia and macrophages. Adv. Exp. Med. Biol. 527, 105–112. 10.1007/978-1-4615-0135-0_12 15206722

[B45] Heyes M. P. Nowak T. S. Jr. (1990). Delayed increases in regional brain quinolinic acid follow transient ischemia in the gerbil. J. Cereb. Blood Flow. Metab. 10 (5), 660–667. 10.1038/jcbfm.1990.119 1696582

[B46] Hilkens N. A. Casolla B. Leung T. W. de Leeuw F.-E. (2024). Stroke. Lancet 403 (10446), 2820–2836. 10.1016/S0140-6736(24)00642-1 38759664

[B47] Hu D. Liu J. Yu W. Li C. Huang L. Mao W. (2023). Tryptophan intake, not always the more the better. Front. Nutr. 10, 1140054. 10.3389/fnut.2023.1140054 37113297 PMC10128863

[B48] Huang Y. Zhao M. Chen X. Zhang R. Le A. Hong M. (2023). Tryptophan metabolism in central nervous system diseases: pathophysiology and potential therapeutic strategies. Aging Dis. 14 (3), 858–878. 10.14336/ad.2022.0916 37191427 PMC10187711

[B50] Ivanov I. I. Atarashi K. Manel N. Brodie E. L. Shima T. Karaoz U. (2009). Induction of intestinal Th17 cells by segmented filamentous bacteria. Cell 139 (3), 485–498. 10.1016/j.cell.2009.09.033 19836068 PMC2796826

[B51] Jackman K. A. Brait V. H. Wang Y. Maghzal G. J. Ball H. J. McKenzie G. (2011). Vascular expression, activity and function of indoleamine 2,3-dioxygenase-1 following cerebral ischaemia–reperfusion in mice. Naunyn-Schmiedeberg's Archives Pharmacol. 383 (5), 471–481. 10.1007/s00210-011-0611-4 21359968

[B52] Jeong J. J. Woo J. Y. Kim K. A. Han M. J. Kim D. H. (2015). Lactobacillus pentosus var. plantarum C29 ameliorates age-dependent memory impairment in Fischer 344 rats. Lett. Appl. Microbiol. 60 (4), 307–314. 10.1111/lam.12393 25598393

[B53] Kalaria R. N. Akinyemi R. Ihara M. (2016). Stroke injury, cognitive impairment and vascular dementia. Biochim. Biophys. Acta 1862 (5), 915–925. 10.1016/j.bbadis.2016.01.015 26806700 PMC4827373

[B54] Kashyap P. C. Marcobal A. Ursell L. K. Larauche M. Duboc H. Earle K. A. (2013). Complex interactions among diet, gastrointestinal transit, and gut microbiota in humanized mice. Gastroenterology 144 (5), 967–977. 10.1053/j.gastro.2013.01.047 23380084 PMC3890323

[B55] Kelly J. R. Borre Y. C O. B. Patterson E. El Aidy S. Deane J. (2016). Transferring the blues: depression-associated gut microbiota induces neurobehavioural changes in the rat. J. Psychiatr. Res. 82, 109–118. 10.1016/j.jpsychires.2016.07.019 27491067

[B56] Kennedy P. J. Cryan J. F. Dinan T. G. Clarke G. (2017). Kynurenine pathway metabolism and the microbiota-gut-brain axis. Neuropharmacology 112 (Pt B), 399–412. 10.1016/j.neuropharm.2016.07.002 27392632

[B57] Ketelhuth D. F. J. (2019). The immunometabolic role of indoleamine 2,3-dioxygenase in atherosclerotic cardiovascular disease: immune homeostatic mechanisms in the artery wall. Cardiovasc Res. 115 (9), 1408–1415. 10.1093/cvr/cvz067 30847484

[B58] Kigerl K. A. Hall J. C. Wang L. Mo X. Yu Z. Popovich P. G. (2016). Gut dysbiosis impairs recovery after spinal cord injury. J. Exp. Med. 213 (12), 2603–2620. 10.1084/jem.20151345 27810921 PMC5110012

[B59] Klein C. Roussel G. Brun S. Rusu C. Patte-Mensah C. Maitre M. (2018). 5-HIAA induces neprilysin to ameliorate pathophysiology and symptoms in a mouse model for Alzheimer's disease. Acta Neuropathol. Commun. 6 (1), 136. 10.1186/s40478-018-0640-z 30537985 PMC6290545

[B60] Krishnan S. Ding Y. Saedi N. Choi M. Sridharan G. V. Sherr D. H. (2018). Gut microbiota-derived tryptophan metabolites modulate inflammatory response in hepatocytes and macrophages. Cell Rep. 23 (4), 1099–1111. 10.1016/j.celrep.2018.03.109 29694888 PMC6392449

[B61] Lee J. C. Tae H. J. Cho G. S. Kim I. H. Ahn J. H. Park J. H. (2015). Ischemic preconditioning protects neurons from damage and maintains the immunoreactivity of kynurenic acid in the gerbil hippocampal CA1 region following transient cerebral ischemia. Int. J. Mol. Med. 35 (6), 1537–1544. 10.3892/ijmm.2015.2171 25872573 PMC4432926

[B62] Lee J.-H. Lee J. (2010). Indole as an intercellular signal in microbial communities. FEMS Microbiol. Rev. 34 (4), 426–444. 10.1111/j.1574-6976.2009.00204.x 20070374

[B63] Lee J. H. Wood T. K. Lee J. (2015). Roles of indole as an interspecies and interkingdom signaling molecule. Trends Microbiol. 23 (11), 707–718. 10.1016/j.tim.2015.08.001 26439294

[B64] Li N. Wang Q. Wang Y. Sun A. Lin Y. Jin Y. (2018). Oral probiotics ameliorate the behavioral deficits induced by chronic mild stress in mice via the gut microbiota-inflammation Axis. Front. Behav. Neurosci. 12, 266. 10.3389/fnbeh.2018.00266 30459574 PMC6232506

[B65] Li Q. Lan X. Han X. Durham F. Wan J. Weiland A. (2021). Microglia-derived interleukin-10 accelerates post-intracerebral hemorrhage hematoma clearance by regulating CD36. Brain Behav. Immun. 94, 437–457. 10.1016/j.bbi.2021.02.001 33588074 PMC8058329

[B66] Li S. Zhao X. Lin F. Ni X. Liu X. Kong C. (2022). Gut flora mediates the rapid tolerance of electroacupuncture on ischemic stroke by activating melatonin receptor through regulating indole-3-propionic acid. Am. J. Chin. Med. 50 (4), 979–1006. 10.1142/s0192415x22500409 35475976

[B67] Li X. Chen D. Chen X. Jiang C. Guo Y. Hang J. (2024). Study on the correlation between serum indole-3-propionic acid levels and the progression and prognosis of acute ischemic stroke. J. Stroke Cerebrovasc. Dis. 33 (6), 107680. 10.1016/j.jstrokecerebrovasdis.2024.107680 38508478

[B68] Lian Z. Xu Y. Wang C. Chen Y. Yuan L. Liu Z. (2023). Gut microbiota-derived melatonin from Puerariae Lobatae Radix-resistant starch supplementation attenuates ischemic stroke injury via a positive microbial co-occurrence pattern. Pharmacol. Res. 190, 106714. 10.1016/j.phrs.2023.106714 36863429

[B69] Liaqat H. Parveen A. Kim S. Y. (2022). Neuroprotective natural products' regulatory effects on depression via gut-brain Axis targeting tryptophan. Nutrients 14 (16), 3270. 10.3390/nu14163270 36014776 PMC9413544

[B70] Lim C. K. Fernández-Gomez F. J. Braidy N. Estrada C. Costa C. Costa S. (2017). Involvement of the kynurenine pathway in the pathogenesis of Parkinson's disease. Prog. Neurobiol. 155, 76–95. 10.1016/j.pneurobio.2015.12.009 27072742

[B71] Liu Y. Liang X. Dong W. Fang Y. Lv J. Zhang T. (2018). Tumor-repopulating cells induce PD-1 expression in CD8(+) T cells by transferring kynurenine and AhR activation. Cancer Cell 33 (3), 480–494.e7. 10.1016/j.ccell.2018.02.005 29533786

[B72] Liu Y. Zhao P. Cai Z. He P. Wang J. He H. (2024). Buqi-Huoxue-Tongnao decoction drives gut microbiota-derived indole lactic acid to attenuate ischemic stroke via the gut-brain axis. Chin. Med. 19 (1), 126. 10.1186/s13020-024-00991-1 39278929 PMC11403783

[B73] Ma N. He T. Johnston L. J. Ma X. (2020). Host-microbiome interactions: the aryl hydrocarbon receptor as a critical node in tryptophan metabolites to brain signaling. Gut Microbes 11 (5), 1203–1219. 10.1080/19490976.2020.1758008 32401136 PMC7524279

[B74] Mackowiak B. Wang H. (2016). Mechanisms of xenobiotic receptor activation: direct vs. indirect. Biochim. Biophys. Acta 1859 (9), 1130–1140. 10.1016/j.bbagrm.2016.02.006 26877237 PMC4975672

[B75] Mangin G. Poittevin M. Charriaut-Marlangue C. Giannesini C. Merkoulova-Rainon T. Kubis N. (2019). Glatiramer acetate reduces infarct volume in diabetic mice with cerebral ischemia and prevents long-term memory loss. Brain Behav. Immun. 80, 315–327. 10.1016/j.bbi.2019.04.009 30953775

[B76] Manzella C. Singhal M. Alrefai W. A. Saksena S. Dudeja P. K. Gill R. K. (2018). Serotonin is an endogenous regulator of intestinal CYP1A1 via AhR. Sci. Rep. 8 (1), 6103. 10.1038/s41598-018-24213-5 29666456 PMC5904159

[B77] Marin I. A. Goertz J. E. Ren T. Rich S. S. Onengut-Gumuscu S. Farber E. (2017). Microbiota alteration is associated with the development of stress-induced despair behavior. Sci. Rep. 7, 43859. 10.1038/srep43859 28266612 PMC5339726

[B78] Martin C. R. Osadchiy V. Kalani A. Mayer E. A. (2018). The brain-gut-microbiome Axis. Cell Mol. Gastroenterol. Hepatol. 6 (2), 133–148. 10.1016/j.jcmgh.2018.04.003 30023410 PMC6047317

[B79] MartInez-Coria H. Arrieta-Cruz I. Cruz M. E. López-Valdés H. E. (2021). Physiopathology of ischemic stroke and its modulation using memantine: evidence from preclinical stroke. Neural Regen. Res. 16 (3), 433–439. 10.4103/1673-5374.293129 32985462 PMC7996012

[B80] Martin-Gallausiaux C. Larraufie P. Jarry A. Béguet-Crespel F. Marinelli L. Ledue F. (2018). Butyrate produced by commensal bacteria down-regulates indolamine 2,3-dioxygenase 1 (Ido-1) expression via a dual mechanism in human intestinal epithelial cells. Front. Immunol. 9, 2838. 10.3389/fimmu.2018.02838 30619249 PMC6297836

[B81] Mawe G. M. Hoffman J. M. (2013). Serotonin signalling in the gut--functions, dysfunctions and therapeutic targets. Nat. Rev. Gastroenterol. Hepatol. 10 (8), 473–486. 10.1038/nrgastro.2013.105 23797870 PMC4048923

[B82] Mayer E. A. Nance K. Chen S. (2022). The gut-brain Axis. Annu. Rev. Med. 73, 439–453. 10.1146/annurev-med-042320-014032 34669431

[B83] McLean P. G. Borman R. A. Lee K. (2007). 5-HT in the enteric nervous system: gut function and neuropharmacology. Trends Neurosci. 30 (1), 9–13. 10.1016/j.tins.2006.11.002 17126921

[B84] Mo X. Pi L. Yang J. Xiang Z. Tang A. (2014). Serum indoleamine 2,3-dioxygenase and kynurenine aminotransferase enzyme activity in patients with ischemic stroke. J. Clin. Neurosci. 21 (3), 482–486. 10.1016/j.jocn.2013.08.020 24412293

[B85] Mu C. Yang Y. Zhu W. (2016). Gut microbiota: the brain peacekeeper. Front. Microbiol. 7, 345. 10.3389/fmicb.2016.00345 27014255 PMC4794499

[B86] Peesh P. Blasco-Conesa M. P. El Hamamy A. Khan R. Guzman G. U. Honarpisheh P. (2025). Benefits of equilibrium between microbiota- and host-derived ligands of the aryl hydrocarbon receptor after stroke in aged male mice. Nat. Commun. 16 (1), 1767. 10.1038/s41467-025-57014-2 39971928 PMC11839985

[B87] Pernomian L. Duarte-Silva M. de Barros Cardoso C. R. (2020). The aryl hydrocarbon receptor (AHR) as a potential target for the control of intestinal inflammation: insights from an immune and bacteria sensor receptor. Clin. Rev. Allergy Immunol. 59 (3), 382–390. 10.1007/s12016-020-08789-3 32279195

[B88] Plitman E. Iwata Y. Caravaggio F. Nakajima S. Chung J. K. Gerretsen P. (2017). Kynurenic acid in schizophrenia: a systematic review and meta-analysis. Schizophr. Bull. 43 (4), 764–777. 10.1093/schbul/sbw221 28187219 PMC5472151

[B89] Prinz M. Masuda T. Wheeler M. A. Quintana F. J. (2021). Microglia and central nervous system-associated macrophages-from origin to disease modulation. Annu. Rev. Immunol. 39, 251–277. 10.1146/annurev-immunol-093019-110159 33556248 PMC8085109

[B90] Qian C. Yang C. Lu M. Bao J. Shen H. Deng B. (2021). Activating AhR alleviates cognitive deficits of Alzheimer's disease model mice by upregulating endogenous Aβ catabolic enzyme Neprilysin. Theranostics 11 (18), 8797–8812. 10.7150/thno.61601 34522212 PMC8419060

[B91] Quigley E. M. Craig O. F. (2012). Irritable bowel syndrome; update on pathophysiology and management. Turk J. Gastroenterol. 23 (4), 313–322. 10.4318/tjg.2012.0551 22965501

[B92] Reiter R. J. Mayo J. C. Tan D. X. Sainz R. M. Alatorre-Jimenez M. Qin L. (2016). Melatonin as an antioxidant: under promises but over delivers. J. Pineal Res. 61 (3), 253–278. 10.1111/jpi.12360 27500468

[B93] Ren H. Han R. Chen X. Liu X. Wan J. Wang L. (2020). Potential therapeutic targets for intracerebral hemorrhage-associated inflammation: an update. J. Cereb. Blood Flow. Metab. 40 (9), 1752–1768. 10.1177/0271678x20923551 32423330 PMC7446569

[B94] Roager H. M. Licht T. R. (2018). Microbial tryptophan catabolites in health and disease. Nat. Commun. 9 (1), 3294. 10.1038/s41467-018-05470-4 30120222 PMC6098093

[B95] Rothhammer V. Borucki D. M. Tjon E. C. Takenaka M. C. Chao C. C. Ardura-Fabregat A. (2018). Microglial control of astrocytes in response to microbial metabolites. Nature 557 (7707), 724–728. 10.1038/s41586-018-0119-x 29769726 PMC6422159

[B96] Rothhammer V. Mascanfroni I. D. Bunse L. Takenaka M. C. Kenison J. E. Mayo L. (2016). Type I interferons and microbial metabolites of tryptophan modulate astrocyte activity and central nervous system inflammation via the aryl hydrocarbon receptor. Nat. Med. 22 (6), 586–597. 10.1038/nm.4106 27158906 PMC4899206

[B97] Rzemieniec J. Castiglioni L. Gelosa P. Muluhie M. Mercuriali B. Sironi L. (2021). Nuclear receptors in myocardial and cerebral ischemia-mechanisms of action and therapeutic strategies. Int. J. Mol. Sci. 22 (22), 12326. 10.3390/ijms222212326 34830207 PMC8617737

[B98] Sadanandan N. Cozene B. Cho J. Park Y. J. Saft M. Gonzales-Portillo B. (2020). Melatonin-A potent therapeutic for stroke and stroke-related dementia. Antioxidants (Basel) 9 (8), 672. 10.3390/antiox9080672 32731545 PMC7463751

[B99] Salminen A. (2023). Activation of aryl hydrocarbon receptor (AhR) in Alzheimer's disease: role of tryptophan metabolites generated by gut host-microbiota. J. Mol. Med. Berl. 101 (3), 201–222. 10.1007/s00109-023-02289-5 36757399 PMC10036442

[B100] Sanders M. E. Merenstein D. J. Reid G. Gibson G. R. Rastall R. A. (2019). Probiotics and prebiotics in intestinal health and disease: from biology to the clinic. Nat. Rev. Gastroenterol. Hepatol. 16 (10), 605–616. 10.1038/s41575-019-0173-3 31296969

[B101] Savitz J. (2020). The kynurenine pathway: a finger in every pie. Mol. Psychiatry 25 (1), 131–147. 10.1038/s41380-019-0414-4 30980044 PMC6790159

[B102] Seo S. K. Kwon B. (2023). Immune regulation through tryptophan metabolism. Exp. Mol. Med. 55 (7), 1371–1379. 10.1038/s12276-023-01028-7 37394584 PMC10394086

[B103] Simunkova M. Alwasel S. H. Alhazza I. M. Jomova K. Kollar V. Rusko M. (2019). Management of oxidative stress and other pathologies in Alzheimer's disease. Arch. Toxicol. 93 (9), 2491–2513. 10.1007/s00204-019-02538-y 31440798

[B104] Song X. Zhu S. Shi P. Liu Y. Shi Y. Levin S. D. (2011). IL-17RE is the functional receptor for IL-17C and mediates mucosal immunity to infection with intestinal pathogens. Nat. Immunol. 12 (12), 1151–1158. 10.1038/ni.2155 21993849

[B105] Sun J. Wang F. Hong G. Pang M. Xu H. Li H. (2016). Antidepressant-like effects of sodium butyrate and its possible mechanisms of action in mice exposed to chronic unpredictable mild stress. Neurosci. Lett. 618, 159–166. 10.1016/j.neulet.2016.03.003 26957230

[B106] Sun M. Ma N. He T. Johnston L. J. Ma X. (2020). Tryptophan (Trp) modulates gut homeostasis via aryl hydrocarbon receptor (AhR). Crit. Rev. Food Sci. Nutr. 60 (10), 1760–1768. 10.1080/10408398.2019.1598334 30924357

[B107] Swanson K. V. Deng M. Ting J. P. (2019). The NLRP3 inflammasome: molecular activation and regulation to therapeutics. Nat. Rev. Immunol. 19 (8), 477–489. 10.1038/s41577-019-0165-0 31036962 PMC7807242

[B108] Turner E. H. Loftis J. M. Blackwell A. D. (2006). Serotonin a la carte: supplementation with the serotonin precursor 5-hydroxytryptophan. Pharmacol. Ther. 109 (3), 325–338. 10.1016/j.pharmthera.2005.06.004 16023217

[B109] Vécsei L. Szalárdy L. Fülöp F. Toldi J. (2013). Kynurenines in the CNS: recent advances and new questions. Nat. Rev. Drug Discov. 12 (1), 64–82. 10.1038/nrd3793 23237916

[B110] Veldhoen M. Hirota K. Westendorf A. M. Buer J. Dumoutier L. Renauld J. C. (2008). The aryl hydrocarbon receptor links TH17-cell-mediated autoimmunity to environmental toxins. Nature 453 (7191), 106–109. 10.1038/nature06881 18362914

[B111] Vergun O. Keelan J. Khodorov B. I. Duchen M. R. (1999). Glutamate-induced mitochondrial depolarisation and perturbation of calcium homeostasis in cultured rat hippocampal neurones. J. Physiol. 519 Pt 2 (Pt 2), 451–466. 10.1111/j.1469-7793.1999.0451m.x PMC2269520 10457062

[B112] Vujkovic-Cvijin I. Dunham R. M. Iwai S. Maher M. C. Albright R. G. Broadhurst M. J. (2013). Dysbiosis of the gut microbiota is associated with HIV disease progression and tryptophan catabolism. Sci. Transl. Med. 5 (193), 193ra91. 10.1126/scitranslmed.3006438 PMC4094294 23843452

[B113] Walstab J. Wohlfarth C. Hovius R. Schmitteckert S. Röth R. Lasitschka F. (2014). Natural compounds boldine and menthol are antagonists of human 5-HT3 receptors: implications for treating gastrointestinal disorders. Neurogastroenterol. Motil. 26 (6), 810–820. 10.1111/nmo.12334 24708203

[B114] Wang D. Wu J. Zhu P. Xie H. Lu L. Bai W. (2022). Tryptophan-rich diet ameliorates chronic unpredictable mild stress induced depression- and anxiety-like behavior in mice: the potential involvement of gut-brain axis. Food Res. Int. 157, 111289. 10.1016/j.foodres.2022.111289 35761597

[B115] Wang J. Gao Y. Yuan Y. Wang H. Wang Z. Zhang X. (2024). Th17 cells and IL-17a in ischemic stroke. Mol. Neurobiol. 61 (4), 2411–2429. 10.1007/s12035-023-03723-y 37884768 PMC10973033

[B116] Wang J. Peng Y. Liu Y. Lian Z. Cai Z. Chen Y. (2025). Indole lactic acid derived from Akkermansia muciniphila activates the aryl hydrocarbon receptor to inhibit ferroptosis in ischemic stroke. Free Radic. Biol. Med. 234, 113–130. 10.1016/j.freeradbiomed.2025.04.020 40246252

[B117] Wei G. Z. Martin K. A. Xing P. Y. Agrawal R. Whiley L. Wood T. K. (2021). Tryptophan-metabolizing gut microbes regulate adult neurogenesis via the aryl hydrocarbon receptor. Proc. Natl. Acad. Sci. U. S. A. 118 (27), e2021091118. 10.1073/pnas.2021091118 34210797 PMC8271728

[B118] Wu C. Wu C. Peng L. Wu M. Li Z. Chen J. (2024). Multi-omics approaches for the understanding of therapeutic mechanism for Huang-Qi-Long-Dan Granule against ischemic stroke. Pharmacol. Res. 205, 107229. 10.1016/j.phrs.2024.107229 38782148

[B119] Wu H. Denna T. H. Storkersen J. N. Gerriets V. A. (2019). Beyond a neurotransmitter: the role of serotonin in inflammation and immunity. Pharmacol. Res. 140, 100–114. 10.1016/j.phrs.2018.06.015 29953943

[B120] Xie X. Wang L. Dong S. Ge S. Zhu T. (2024a). Immune regulation of the gut-brain axis and lung-brain axis involved in ischemic stroke. Neural Regen. Res. 19 (3), 519–528. 10.4103/1673-5374.380869 37721279 PMC10581566

[B121] Xie X. D. Dong S. S. Liu R. J. Shi L. L. Zhu T. (2024b). Mechanism of efferocytosis in determining ischaemic stroke resolution-diving into microglia/macrophage functions and therapeutic modality. Mol. Neurobiol. 61, 7583–7602. 10.1007/s12035-024-04060-4 38409642

[B122] Xie Y. Zou X. Han J. Zhang Z. Feng Z. Ouyang Q. (2022). Indole-3-propionic acid alleviates ischemic brain injury in a mouse middle cerebral artery occlusion model. Exp. Neurol. 353, 114081. 10.1016/j.expneurol.2022.114081 35405119

[B123] Xue C. Li G. Zheng Q. Gu X. Shi Q. Su Y. (2023). Tryptophan metabolism in health and disease. Cell Metab. 35 (8), 1304–1326. 10.1016/j.cmet.2023.06.004 37352864

[B124] Yang J. Z. Zhang K. K. Shen H. W. Liu Y. Li X. W. Chen L. J. (2023). Sigma-1 receptor knockout disturbs gut microbiota, remodels serum metabolome, and exacerbates isoprenaline-induced heart failure. Front. Microbiol. 14, 1255971. 10.3389/fmicb.2023.1255971 37720144 PMC10501138

[B125] Young K. D. Drevets W. C. Dantzer R. Teague T. K. Bodurka J. Savitz J. (2016). Kynurenine pathway metabolites are associated with hippocampal activity during autobiographical memory recall in patients with depression. Brain Behav. Immun. 56, 335–342. 10.1016/j.bbi.2016.04.007 27091600 PMC4917447

[B126] Zelante T. Iannitti R. G. Cunha C. De Luca A. Giovannini G. Pieraccini G. (2013). Tryptophan catabolites from microbiota engage aryl hydrocarbon receptor and balance mucosal reactivity via interleukin-22. Immunity 39 (2), 372–385. 10.1016/j.immuni.2013.08.003 23973224

[B127] Zhang H. Hui X. Wang Y. Wang Y. Lu X. (2022). Angong Niuhuang Pill ameliorates cerebral ischemia/reperfusion injury in mice partly by restoring gut microbiota dysbiosis. Front. Pharmacol. Volume, 1001422–1002022. 10.3389/fphar.2022.1001422 PMC9520595 36188565

[B128] Zhang H. Jin B. You X. Yi P. Guo H. Niu L. (2023). Pharmacodynamic advantages and characteristics of traditional Chinese medicine in prevention and treatment of ischemic stroke. Chin. Herb. Med. 15 (4), 496–508. 10.1016/j.chmed.2023.09.003 38094018 PMC10715896

[B129] Zhang J. Zhu S. Ma N. Johnston L. J. Wu C. Ma X. (2021a). Metabolites of microbiota response to tryptophan and intestinal mucosal immunity: a therapeutic target to control intestinal inflammation. Med. Res. Rev. 41 (2), 1061–1088. 10.1002/med.21752 33174230

[B130] Zhang Q. Liao Y. Liu Z. Dai Y. Li Y. Li Y. (2021b). Interleukin-17 and ischaemic stroke. Immunology 162 (2), 179–193. 10.1111/imm.13265 32935861 PMC7808154

[B131] Zhao W. Wu C. Stone C. Ding Y. Ji X. (2020). Treatment of intracerebral hemorrhage: current approaches and future directions. J. Neurol. Sci. 416, 117020. 10.1016/j.jns.2020.117020 32711191

[B132] Zhu L. Wei T. Gao J. Chang X. He H. Miao M. (2015). Salidroside attenuates lipopolysaccharide (LPS) induced serum cytokines and depressive-like behavior in mice. Neurosci. Lett. 606, 1–6. 10.1016/j.neulet.2015.08.025 26300543

[B133] Zhu T. Dong S. Qin N. Liu R. Shi L. Wan Q. (2024). Dl-3-n-butylphthalide attenuates cerebral ischemia/reperfusion injury in mice through AMPK-mediated mitochondrial fusion. Front. Pharmacol. 15, 1357953. 10.3389/fphar.2024.1357953 38455957 PMC10917971

